# N-MYC DOWN-REGULATED-LIKE Proteins Regulate Meristem Initiation by Modulating Auxin Transport and *MAX2* Expression

**DOI:** 10.1371/journal.pone.0077863

**Published:** 2013-11-04

**Authors:** Yashwanti Mudgil, Sanjay Ghawana, Alan M. Jones

**Affiliations:** 1 Department of Botany, University of Delhi, Delhi, India; 2 Departments of Biology and Pharmacology, University of North Carolina at Chapel Hill, Chapel Hill, North Carolina, United States of America; Universidad Miguel Hernández de Elche, Spain

## Abstract

**Background:**

N-MYC DOWN-REGULATED-LIKE (NDL) proteins interact with the Gβ subunit (AGB1) of the heterotrimeric G protein complex and play an important role in AGB1-dependent regulation of lateral root formation by affecting root auxin transport, auxin gradients and the steady-state levels of mRNA encoding the *PIN-FORMED 2* and *AUXIN 1* auxin transport facilitators. Auxin transport in aerial tissue follows different paths and utilizes different transporters than in roots; therefore, in the present study, we analyzed whether NDL proteins play an important role in AGB1-dependent, auxin-mediated meristem development.

**Methodology/Principal Findings:**

Expression levels of *NDL* gene family members need to be tightly regulated, and altered expression (both over-expression and down-regulation) confers ectopic growth. Over-expression of *NDL1* disrupts vegetative and reproductive organ development. Reduced expression of the *NDL* gene family members results in asymmetric leaf emergence, twinning of rosette leaves, defects in leaf formation, and abnormal silique distribution. Reduced expression of the *NDL* genes in the *agb1-2* (null allele) mutant rescues some of the abnormal phenotypes, such as silique morphology, silique distribution, and peduncle angle, suggesting that proper levels of NDL proteins are maintained by AGB1. We found that all of these abnormal aerial phenotypes due to altered *NDL* expression were associated with increases in basipetal auxin transport, altered auxin maxima and altered *MAX2* expression within the inflorescence stem.

**Conclusion/Significance:**

NDL proteins, together with AGB1, act as positive regulators of meristem initiation and branching. AGB1 and NDL1 positively regulate basipetal inflorescence auxin transport and modulate *MAX2* expression in shoots, which in turn regulates organ and lateral meristem formation by the establishment and maintenance of auxin gradients.

## Introduction

Shoot architecture maintenance is important for plants to adapt to shifting conditions. It is a complex and finely tuned process regulated by the interplay of environmental and endogenous signals, such as plant hormones. Hormonal control of shoot branching is mediated by the interplay of auxins, cytokinins and strigolactones [1]–[6]. Auxins inhibit bud outgrowth by positively regulating the biosynthesis of strigolactones (inhibitors of branching) [1], [5], [7], [8]. Auxin-mediated regulation of strigolactones occurs through the upregulation of genes encoding enzymes that function in strigolactone synthesis, such as *MORE AXILLARY GROWTH1*, *3* and *4* (*MAX1, MAX3* and *MAX4*) [8]–[10]. Cytokinins are positive regulators of branching [3], [11]–[14], and auxin regulates the local amount of cytokinin by regulating the expression of genes involved in cytokinin biosynthesis and metabolism [1], [2], [14]–[16].

During early *Arabidopsis* development, many tissues (e.g., shoot apical meristems [SAM], cotyledons, leaves and root apical meristems [RAM]) synthesize auxin, and the coordination of global auxin synthesis, auxin transport, and local auxin catabolism act in concert to form local auxin gradients, which are critical for normal growth and development. [17]–[27].

It is well established in *Arabidopsis* that organ formation is preceded by the establishment of auxin maxima where primordia will form [28]. Auxin gradients are created, in part, by the family of membrane-localized PIN-formed (PIN) proteins [23], [29]–[32]. PIN proteins regulate auxin flux in both aerial and underground organs, and the concomitant establishment of local auxin gradients/maxima are required for the formation of all plant organs [33], [34].

The phyllotaxis of lateral organs around the central axis is regulated by active auxin transport and the resulting locations of auxin maxima [35]–[38]. Heterotrimeric G protein signaling components, especially AGB1, are negative regulators of auxin transport, and auxin-induced cell division [39], [40]. We have previously shown that NDL proteins physically interact with AGB1, and these proteins act in both a concerted and antagonistic manner to regulate auxin transport streams in roots by controlling, in part, the levels of auxin transport facilitators [40].

Here, we show that the abnormal aerial phenotypes due to altered expression of *NDL* gene family members in the Col-0 and *agb1* mutant backgrounds, such as aberrant branching and altered organ initiation, shape and arrangement, are the result of altered auxin transport and, in part, altered *MAX2* expression levels. Specifically: 1) NDL1 is excluded from/peripherally localized in the meristem and acts as a positive regulator of meristem initiation and shoot branching in a G protein-dependent manner; 2) changes in NDL protein steady-state levels disrupt vegetative growth, the reproductive phase, organ shape and patterning and terminal differentiation of the floral meristem; 3) NDL proteins modulate basipetal auxin transport in the inflorescence stem and local auxin gradients in shoots; and 4) *NDL1* and *AGB1* modulate *MAX2* expression levels in an NDL1-dependent manner.

## Results

### NDL1 Localization at the Vegetative and Reproductive Meristems

**Figure 1:**
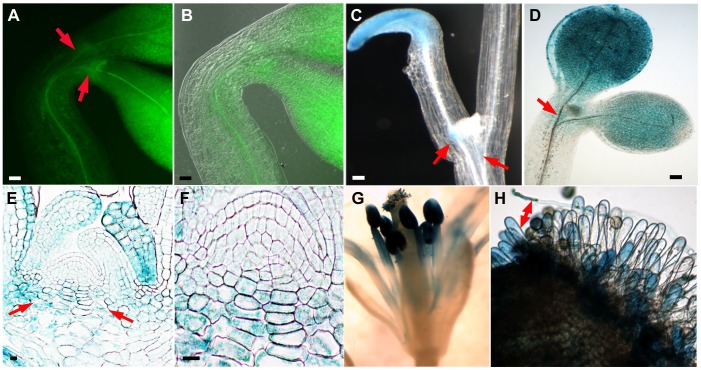
GUS and GFP reporter gene analyses of *NDL1* expression around the vegetative and reproductive SAMs in *Arabidopsis* seedlings. (**A**) NDL1-GFP localization in three-day-old etiolated seedlings. GFP fluorescence is detectable in the vicinity of the SAM. (**B**) Panel A with an overlay of the DIC image. (**C**) and (**D**) GUS histochemical staining in eight-day-old etiolated seedlings (**C**) and in ten-day-old seedlings grown in the light (**D**). GUS staining is not detectable in the SAM. (**E**) GUS histochemical staining in a longitudinal section of the vegetative SAM in etiolated seedlings. (**F**) Enlarged view of the SAM from panel E. (**G**) GUS staining in a mature stamen. (**H**) Papillar cells showing GUS staining upon pollen germination (the red double-ended arrow points to a germinating pollen tube). Scale bars = 50 µm, Red arrows in panels A, C, D and E indicate cell zones at the periphery of the SAM.

**Figure 2:**
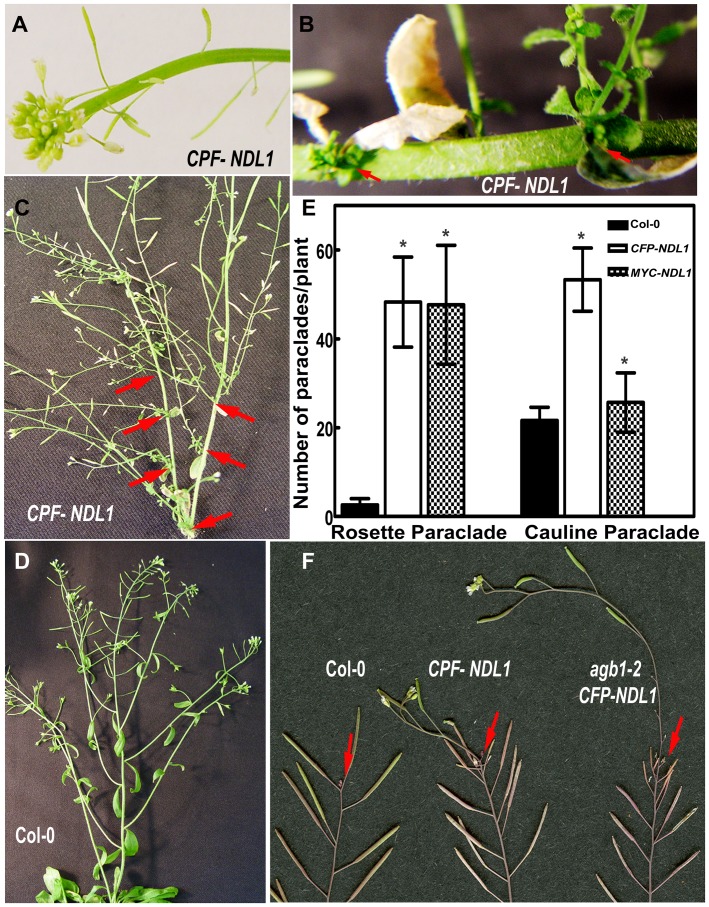
Vegetative growth phenotypes of *NDL1* over-expression lines. *Arabidopsis* plants over-expressing *NDL1* under the control of the 35 S promoter were used for phenotypic analyses. (**A**) Inflorescence stems show fasciation. (**B**) Nascent rosettes emerging from the axils of senescing leaves (red arrows). (**C**) Shoots of mature plants contain additional rosettes and cauline paraclades (red arrows). (**D**) Vegetative growth of a Col-0 wild-type control plant. (**E**) Quantification of rosette and cauline paraclades in Col-0 wild-type and 35 S-*NDL1* over-expression lines. Fifteen to twenty independent plants were analyzed for each genotype, and error bars represent SE. Student's t test results are based on differences between wild type and the indicated genotype, and asterisks indicate that P<0.05. (**F**) “Shoot upon shoot” phenotype of a Col-0 wild-type plant and an *agb1-2* mutant over-expressing *NDL1* under the control of the endogenous *NDL1* promoter. Red arrows point to the origins of the nascent shoots.

*In situ* localization of the NDL1 protein was indirectly determined by analyzing three independent translational fusion lines containing GUS and GFP (*pNDL:NDL1-GUS*/*GFP*). The NDL1 protein was excluded from the SAM; however, fusion proteins were detectable in the cells flanking the vegetative meristem. Both light-grown and etiolated seedlings during early (three-day-old seedlings, Fig. 1A, red arrows) and later (eight- to ten-day-old seedlings, Fig. 1C and D, red arrows) stages of development showed a similar pattern of NDL1-GUS localization around the SAM. Sagittal sections of the SAM also revealed strong GUS staining in the cells peripheral to the SAM (Fig. 1 E and F). Asymmetrical NDL1-GUS localization was observed, with one cotyledon showing much stronger staining than the other (Fig. 1 and Fig. S1 in File S1). This asymmetry was more severe and frequent in etiolated seedlings compared to light-grown seedlings. As shown in Fig 1C, many dark-grown seedlings had one cotyledon that lagged in expansion, and in these cases this cotyledon showed higher NDL1-GUS/GFP levels (cf. Fig. S1B in File S1). The same delay in expansion was observed, albeit with less severity, in light-grown cotyledon pairs (cf. Fig. 1D and Fig. S1A in File S1). NDL1 localization analysis in mature reproductive meristems showed strong GUS staining in mature flower stamens (Fig. 1G). Germinating pollen exhibited deep staining in the pollen tubes (Fig. 1H), and the papillar cells of the stigmas also showed GUS staining upon pollen landing and germination (Fig. 1H, double ended red arrow). We previously reported detailed GUS staining results for young emerging cotyledons, early rosette/vegetative leaves (epidermis and trichomes), and stamens (Fig. 2 and Supplemental Fig. 3 of [40]).

**Figure 3:**
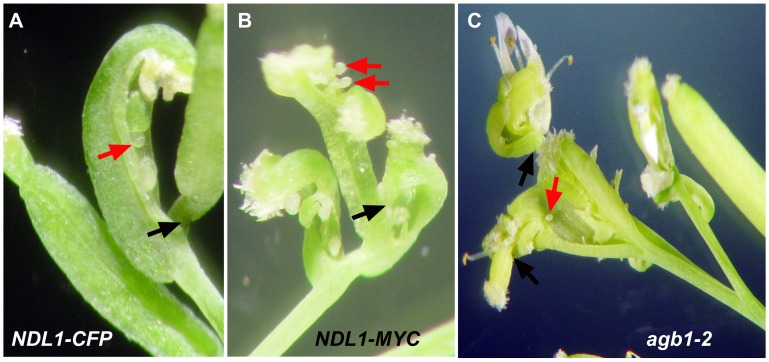
Reproductive growth phenotypes of *NDL1* over-expression lines. Twelve week old *Arabidopsis* plants over-expressing *CFP* and *MYC*-*NDL1* under the control of the 35 S promoter were used for phenotypic analyses. (**A**) Ectopic over-expression of *NDL1* causes a silique to emerge from another silique, which contains an open carpel bearing ovules. (**B**) Open siliques emerging from a single pedicel. (**C**) Shoots of *agb1-2* mutant plants contain abnormal terminal flowers. Red arrows mark open ovules, and black arrows mark pistils emerging from a silique.

### NDL1 is a Positive Regulator of Reproductive and Secondary Vegetative Meristems

Over-expression of *NDL1* using ten independent lines harboring *35* *S:CFP-NDL1* and *35* *S:MYC-NDL1* constructs resulted in ectopic growth of vegetative, and reproductive organs. During primary vegetative growth, approximately 80% of the plants showed stem fasciation (Fig. 2A), and mature plants nearing senescence underwent a secondary burst of vegetative growth (compare Fig. 2B and C at the red arrows to D, Col-0). Ectopic vegetative and reproductive structures originated from the main stem. New rosettes emerged from the axils of senescent, cauline leaves (Fig. 2B, red arrows), giving rise to cauline paraclades. This secondary growth resulted in more rosettes, as well as cauline paraclades, manifesting as a highly branched plant. Over-expression of both constructs resulted in an eighteen-fold increase in rosette paraclades. Plants expressing *CFP-NDL1* showed a two-fold increase in cauline paraclades compared to those expressing *MYC-NDL1*, which showed a subtle but statistically significant increase compared to wild type (Fig. 2E, P<0.05). The terminal ends of these secondary and tertiary cauline paraclades contained cauline leaves and flower buds (Fig. S2A and B, red arrows in File S1). Expression of *NDL1* under its native promoter in the Col-0 and *agb1-2* backgrounds was performed using the *pNDL1:NDL1-GUS* construct, and five independent lines were analyzed. In the Col-0 background, expression of this construct resulted in a burst of vegetative growth when mature plants entered late senescence and manifested as green shoots emerging from the terminal shoot (Fig. 2F, red arrows). These ectopic shoots (Fig. 2F, center inflorescence stem) were longer in the absence of AGB1 (Fig. 2F, far right inflorescence stem).

### Proper Level of NDL and AGB1 are Necessary for Flower Development

We analyzed the flower phenotypes of ten lines ectopically expressing *NDL1* (*35* *S:CFP-NDL1* and *35* *S:MYC-NDL1*). Eighty to 90% of the flowers resulting from the secondary vegetative burst of *NDL1* ectopic expression were abnormal, having an atypical number of flower whorls (Fig. S3 in File S1) with open carpels bearing naked ovules (Fig. 3A and B; red arrows indicate open carpels), multiple carpels fused together (Fig. 3B), and carpels emerging from open siliques (Fig. 3A and B, black arrows). The terminal inflorescence stems of the *agb1-2* mutant also contained flowers with similar abnormalities, although at a lower frequency (∼2–5%) (Fig. 3C).

These phenotypes indicate that new flowers are indefinitely produced within the initial flowers as if stem cells are maintained in the centers of the floral meristems. A similar loss of floral meristem termination was reported for the weaker *agamous* (*ag*) alleles (*ag-4* and *AG-Met-205*). *AG* is the main developmental switch towards floral meristem termination and acts by turning *WUSCHEL* off at stage 6 of flower development [41]–[43].

### NDL and AGB1 Operate Together in Organ Initiation, Shape, and Patterning

**Figure 4:**
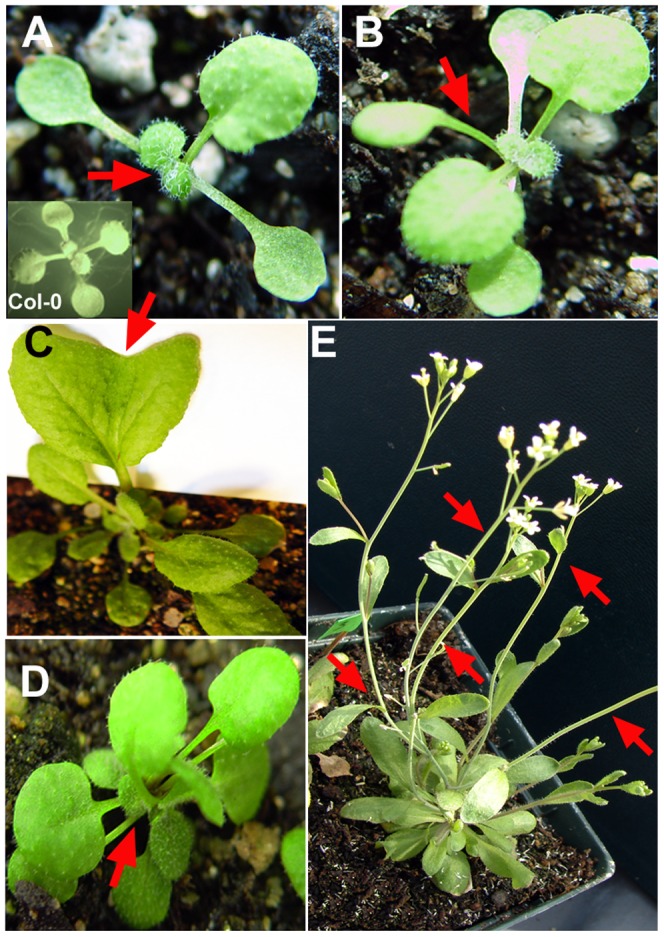
Reduced expression of the *NDL* genes affects vegetative organ development in *Arabidopsis*. Vegetative growth of *ndlM* mutant plants with reduced expression of all three *NDL* genes. (**A**) Altered phyllotactic pattern of *ndlM* mutants and Col-0 wild-type plants (inset) during early stages of development. *ndlM* mutants, but not Col-0 plants, show asymmetrical leaf emergence (the red arrow points to the missing partner of the leaf pair). (**B**) Some *ndlM* mutants form an *Arabidopsis* tricot mutant-like structure (red arrow). (**C**) Leaf phenotype of *ndlM* mutants. Some of the early rosette/vegetative leaves show defects in leaf shape and size. The arrow points to the notch of a heart-shaped leaf with an enlarged lamina. (**D**) More than 80% of *ndlM* mutant plants displayed twinning or the formation of multiple rosettes. The arrow points to the center of a twinned rosette. (**E**) *ndlM* mutant primary shoots emerging from a twinned rosette. The red arrows point to rosette paraclades.

**Figure 5:**
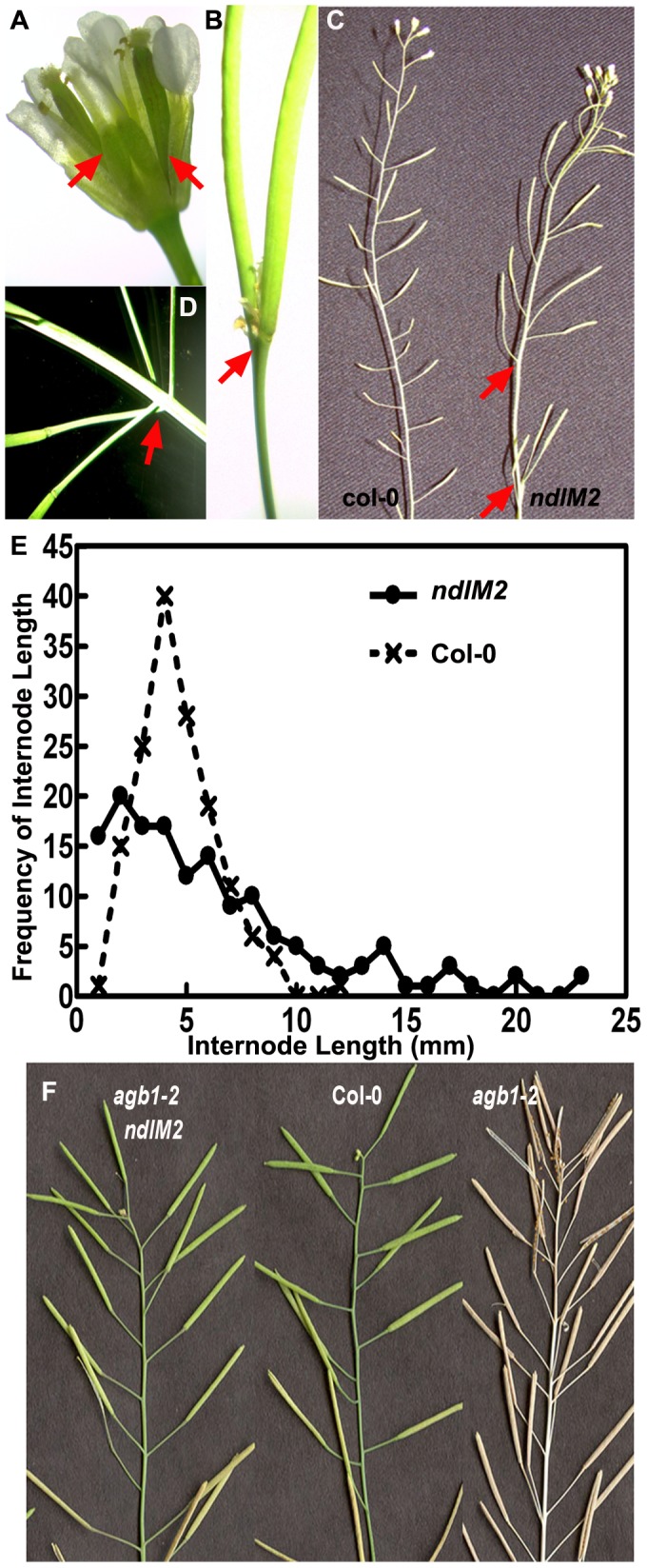
Reduced expression of *NDL* genes affects reproductive organ development in *Arabidopsis*. Phenotypic analyses of reproductive organs from twelve-week-old *ndlM* mutants. (**A**) Twinned flowers with two pistils (red arrows). (**B**) Silique formation from twin flowers of *ndlM* mutants. The red arrow marks the base of the fused pistils. (**C**) Shoots of mature *ndlM* plants show asymmetric silique distribution compared to Col-0 wild-type plants (left). Arrows indicate an abnormally large internode in the *ndlM* plant. (**D**) Internodes of *ndlM* mutants show zones of compaction (arrows). (**E**) Frequencies of internodal lengths between siliques in *ndlM2* mutants compared to Col-0. (**F**) Comparison of silique development in Col-0 wild-type plants (center), *agb1-2* mutants (right) and *agb1-2,ndlM2* mutants (left). Downregulation of the *NDL* genes in the *agb1-2* mutant background rescues some of the abnormal *agb1-2* silique phenotypes such as shape, angle and internode length.

Because *ndl1* knockout mutants did not display developmental defects, and null mutants in the other two *NDL*-like genes were unavailable, a microRNA based approach was used to reduce gene expression of the entire gene family [40]. At least four transgenic lines generated from two different microRNA constructs targeting different region of the mRNAs (*ndlM1* and *ndlM2*) were characterized and found to have similar phenotypes (Fig. 4). Reduced expression of all members of the *NDL* gene family resulted in asymmetric leaf emergence at an early stage of leaf development. Light-grown seedlings often displayed altered leaf phyllotaxis (Fig. 4A and B, arrow). Some of the early leaves were abnormal in shape and size. They had normal petioles, but the lamina showed bifurcation, leading to twinning with independent midveins in both leaf lobes, serrated margins, and folded ends (Fig. 4C and Fig. S4 in File S1). We frequently observed an ancillary rosette fused to the main rosette, and both rosettes shared the central leaves (Fig. 4D; the arrow marks the second rosette). The appearance of these twinned plants became apparent by two weeks. These twinned plants matured, and formed twin or multiple reproductive shoots (Fig. 4E) that bore some twin flowers and siliques (∼5% frequency) (Fig. 5A and B). The majority of the flowers and siliques had normal morphology and size, but their distribution along the stem, and their arrangement were abnormal compared to wild type (Fig. 5C and E, arrows). The range of internode lengths was larger in plants having reduced *NDL* expression compared to wild-type controls (1 mm to 23 mm vs 1 mm to 13 mm) (Fig. 5E). Consequently, multiple siliques sometimes originated from one node or from one small patch on the stem; intermittently, bare regions without siliques also occurred (Fig. 5C and D). Our previous epistasis analysis in roots showed that NDL and AGB1 operate together, and AGB1 is required for NDL1 stability (see Fig. 4 of [40]). In addition, reduced expression of both genes results in some shared defects in flower development and silique shape, angle, and distribution [39], [40]. Therefore, we used the *ndlM2* microRNA to reduce gene expression of the entire *NDL* family in the *agb1-2* background. Five independent *ndlM2,agb1-2* lines were analyzed, and reduced *NDL* expression rescued (Fig. 5F, left) the silique shape and angle, and the internode distance defects of *agb1-2* (Fig. 5F, right) to the wild type phenotypes (Fig. 5F, center).

### NDL1 Activates Dormant Axillary Meristems

**Figure 6:**
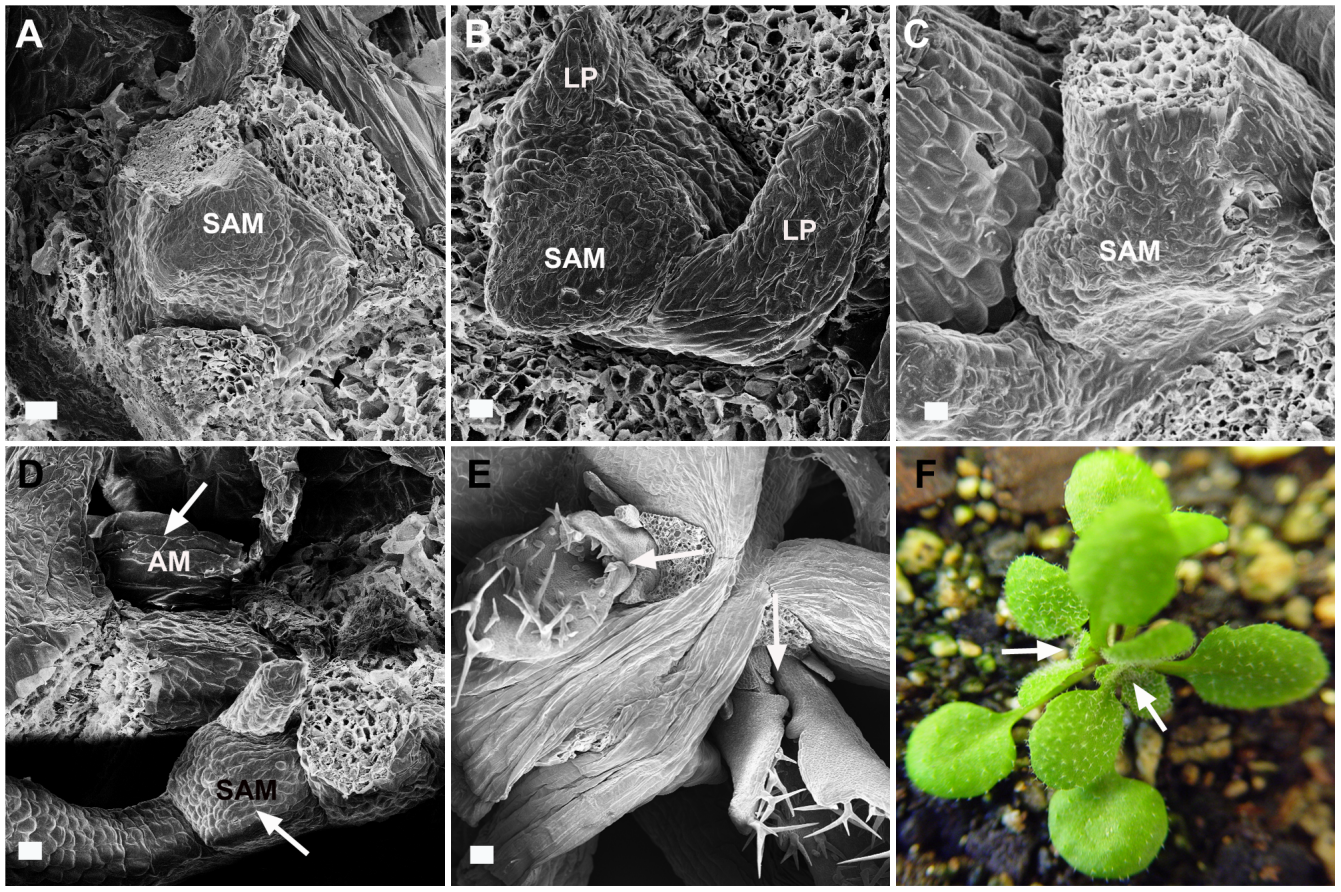
Phenotypic analyses of the shoot apical meristem and the axillary meristem in *ndlM* mutant plants. (**A-E**) Field-emission scanning electron microscopy images of the shoot apical meristem (SAM) and the axillary meristem (AM) in *ndlM* mutants. Developing SAMs were analyzed at various stages of vegetative growth (**A**) Two-day-old plant. (**B**) Four-day-old plant. (**C**) Eight-day-old plant. (**D**) Fourteen-day-old plant. (**E**) Three-week-old plant. The arrows in (D) and (E) indicate the positions of the SAMs and the AMs. (**F**) *ndlM* plant showing twin rosettes. The white arrows point to each rosette head. LP: leaf primordia, (A-E) Scale bars = 20 µm.

To determine the basis of the twin or multiple rosette formation in plants with reduced *NDL* expression, the meristems of two independent lines of *ndlM* mutant plants were imaged during early vegetative growth using field-emission scanning electron microscopy (FESEM) (Fig. 6). Two- to eight-day-old *ndlM* plants formed a single vegetative meristem, giving rise to leaf primordia that were indistinguishable from the wild-type meristem (Fig. 6A–C, showing the SAMs from two-, four- and eight-day-old *ndlM* seedlings, respectively). At a later stage of development, twelve to fourteen-day-old *ndlM* plants showed emergence of an axillary meristem (Fig. 6D; white arrows mark the SAM and the AM). Growth of this new meristem caught up with the primary apical meristem by the third week (Fig. 6E; white arrows indicate the two rosettes), leading to the formation of a twinned rosette in *ndlM* plants (Fig. 6F, early stage of a twinned rosette).

### NDL Proteins and AGB1 Modulate Basipetal Auxin Transport in Inflorescence Stems

**Figure 7:**
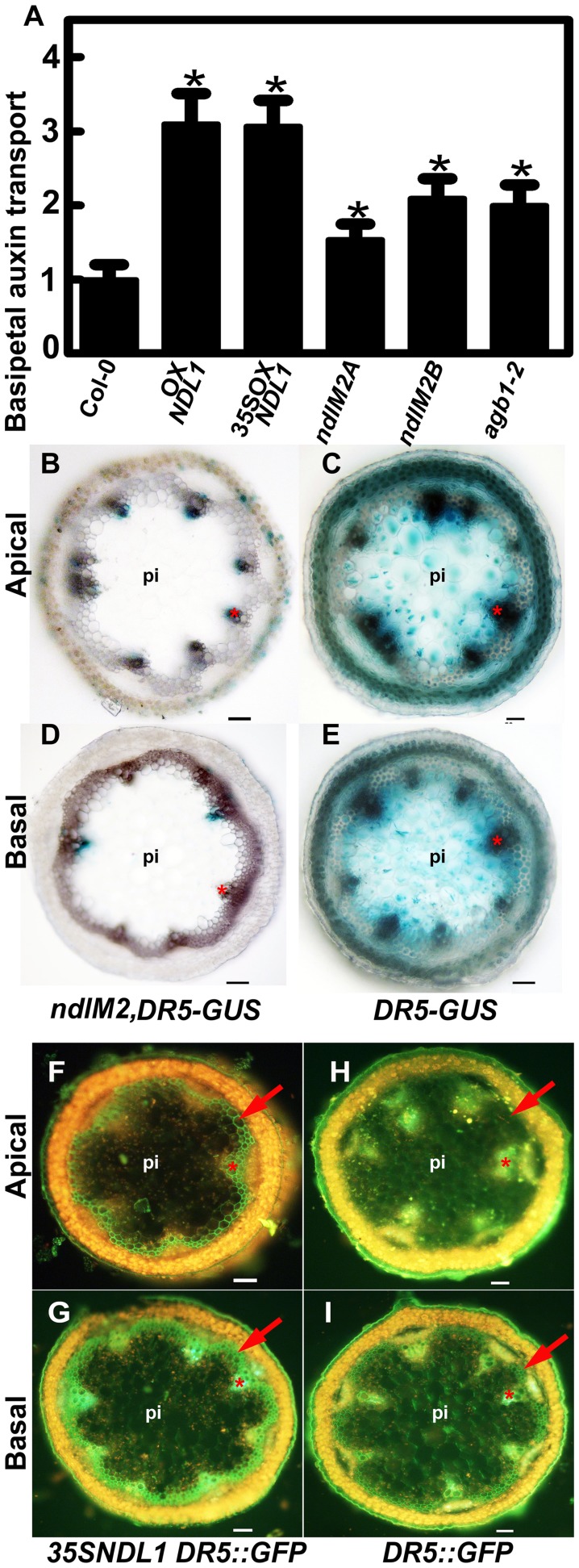
*NDL* expression affects auxin transport in the inflorescence stem and local auxin gradients. (**A**) Measurement of basipetal auxin transport using [^3^H]-IAA in inflorescence stems of *NDL1* over-expression plants and *NDL* knock-down (*ndlM2A and ndlM2B*, two independent *ndlM2* lines) plants in comparison to Col-0 wild type and *agb1* mutant plants. The mean ± SEM values are based on more than 50 shoots per genotype. Student's *t* tests are based on differences between wild type and the indicated genotypes. A confidence level of P<0.05 is indicated by an asterisk. (**B-E**) Histochemical analyses of *DR5:GUS* expression in *NDL* knock-down mutants. GUS staining in apical (**B**) and basal (**D**) stem sections of *ndlM2* mutant lines compared to the corresponding regions of the Col-0 wild-type plants (**C and E**). (**F-I**) Histochemical analyses of *DR5:GFP* expression in *NDL1* over-expressing plants (*35SNDL1*). GUS staining in the apical (**F**) and basal (**G**) stem sections of *35SNDL1* lines compared to the corresponding regions in Col-0 wild-type plants (**H and I**). Scale bars (B to I) = 50 µm, pi = pith. The red arrows and the stars mark the positions of the interfascicular region and the xylem, respectively.

Basipetal auxin movement in stems plays an important role in maintaining apical dominance and inhibiting axillary outgrowth [44]. Consistent with this, we previously showed that NDLs play an important role in AGB1-dependent regulation of lateral root formation by affecting root auxin transport, and auxin gradients. AGB1, a physical partner of NDL1, negatively regulates auxin-induced cell division, and a detailed analysis of *agb1* mutants revealed various vegetative and reproductive defects, indicative of altered auxin patterns [39], [40]. The abnormal aerial phenotypes due to altered *NDL* expression levels (Figs. 2E and 4E) prompted the hypothesis that altered auxin transport and/or distribution is the mechanistic basis for this ectopic, polarized growth. To test this hypothesis, we examined basipetal auxin transport in inflorescence shoots in plants with altered *NDL* expression levels and in the *agb1* mutant (Fig. 7A).

Both ectopic (35 S promoter) and native over-expression (using the *NDL1* promoter) of *NDL1* resulted in at least a three-fold increase in basipetal auxin transport in the inflorescence stem compared to untransformed Col-0 control plants (Fig. 7A). Two independent *ndlM2* microRNA lines (Fig. 7A *ndlM2A and ndlM2B*) both showed an increase in auxin transport, although it was lower compared to plants that over-expressed *NDL1* (maximum of two fold). The *agb1*-*2* single mutant also displayed increased basipetal auxin transport comparable to plants with downregulated *NDL* (Fig. 7A), which corresponds with the phenotypic data. Plants lacking AGB1 or with a sub-optimal level of NDL showed abnormally high auxin transport capacity. We speculate that this increased capacity depletes auxin from some regions of the SAM, thereby activating axillary meristems.

### NDL Proteins are Involved in Establishing Local Auxin Gradients

Because altered levels of NDL cause a significant increase in basipetal inflorescence stem auxin transport (Fig. 7A), we hypothesized that NDL proteins play a role in setting up local auxin gradients in the stem, and therefore modulate the expression of auxin-responsive genes. Furthermore, we propose that the appearance of ectopic auxin maxima underlies the ectopic organ formation observed. We used an auxin-inducible promoter fused with GUS or GFP (DR5:GUS and DR5:GFP) to examine the effects of altered *NDL* expression on the auxin maxima in stems. Four independent *ndlM, DR5:GUS* lines, which had reduced expression of the *NDL* members and carried *DR5:GUS*, showed a substantial decrease in GUS activity/auxin responsiveness in the apical and basal stem vasculature compared to GUS activity in the wild-type background at the same position in the stem (Fig. 7B-E). Histology of the stem vasculature of both genotypes was analyzed by phloroglucinol staining and found to be normal (Fig. S5 in File S1). Analysis of *DR5:GFP* expression in the inflorescence stems of wild type revealed GFP localization in the epidermis, xylem and pith cells of the apical section (Fig. 7H), whereas GFP localization also extended to the interfascicular region, the xylem and the epidermis in *35* *S:NDL1, DR5:GFP* lines over-expressing *NDL1* (Fig. 7F). In the basal stem sections, GFP localization was observed in the interfascicular region as well as in the epidermis, and xylem tissue in the wild-type lines (Fig. 7I). GFP levels were substantially increased in the xylem and the interfascicular region in the inflorescence stems of *NDL1* over-expression lines (Fig. 7G).

### NDL Proteins Affect *MAX2* Expression

**Figure 8:**
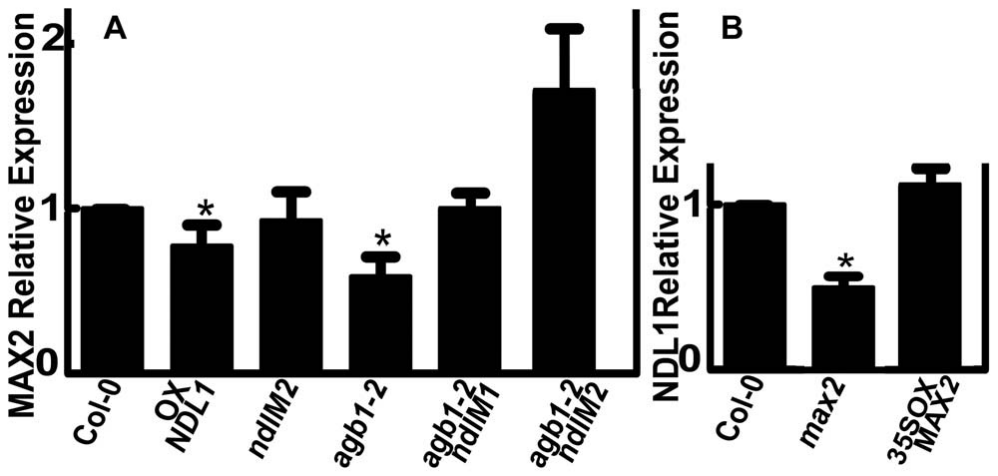
*NDL* and *AGB1* expression affect relative *MAX2* expression levels in flowers. (**A**) qRT-PCR analysis to determine *NDL*- and *AGB1*-dependent relative *MAX2* expression levels. (**B**) qRT-PCR analysis to determine *MAX2*-dependent *NDL1* expression levels. Reactions were performed in triplicate, and three biological replicates were used. Error bars represent the SE. Student's t test results are based on differences between the wild type and the indicated genotypes and are shown as asterisks: *, P<0.05.

Polar auxin transport in shoots requires basally localized PIN1 at the plasma membrane of the xylem parenchyma cells. Strigolactone signaling via MAX2 depletes PIN1 from the plasma membrane of the xylem parenchyma cells in the shoots [45]. We previously found that NDLs function in an AGB1-dependent manner to regulate lateral root formation by affecting auxin transport, and steady-state levels of the mRNA encoding *PIN-FORMED 2* and *AUXIN 1* auxin transport facilitators [40]. In shoots, we also found that alterations in *NDL* and *AGB1* expression lead to increased basipetal auxin transport in aerial shoots, a phenotype similar to the *max2* mutant. These two lines of evidence suggest that NDL may regulate the amount of the MAX2 protein. *NDL1* may act in an auxin-dependent feedback loop to regulate *MAX2* levels; therefore, we determined *MAX2* expression in plants with various *NDL* expression levels, and reciprocally tested the *NDL1* expression level in flowers of the *max2* mutant and a *MAX2* over-expression line using qRT-PCR. Plants expressing *NDL1* under its native promoter, but not those with decreased expression of *NDL*s, showed a 20% reduction in *MAX2* expression in flowers. The absence of AGB1 resulted in a 40% decrease in *MAX2* RNA steady-state levels (Fig. 8A). Down-regulation of *NDL* expression in the absence of AGB1 resulted in wild-type levels of *MAX2* expression in flowers, which is consistent with the phenotype (see Fig. 5F).

In the *max2* mutant background, the *NDL1* expression level was 50% lower than the wild type level, whereas in the *MAX2* over-expression background, the *NDL1* expression level was comparable to the wild-type level (Fig. 8B). These results suggest that NDL and AGB1 proteins function in meristem initiation and shoot branching by directly affecting *MAX2* expression.

## Discussion

NDL proteins interact with G protein signaling components (both AtRGS1 and Gβγ) and regulate root growth by modulating auxin transport and auxin gradients in the root [40]. Auxin patterns established by polar auxin transport are critical throughout plant development, and AGB1 is known to regulate or couple signaling pathways in organs distal to the root [39], [46], [47]. Altering NDL and AGB1 levels confers a number of abnormal aerial phenotypes that likely result from altered auxin patterns or maxima. NDL proteins are important for proper meristem maintenance and, hence, organ initiation, shape, and patterning.

The transition from vegetative to reproductive development is controlled by multiple environmental and endogenous signals. CRABS CLAW (CRC) is a YABBY transcription factor expressed in developing carpel tissue, and it specifically controls the female developmental program. *CRC* expression is activated by *AG* and is present from stage 6 onward [48], [49]. CRC shares AG's function in floral meristem termination, although its activation and function are partially independent of *AG*. Several members of the *YABBY* gene family exhibit complex interactions with meristematic genes, including the *KNOX I* genes *WUSCHEL* and *CLAVATA3* [50]–[53]. These interactions are responsible for CRC's function in floral meristem termination. *YAB2* is the only *YABBY* gene able to rescue *crc*-*1* [54], [55]. The localization of *YABBY* around the SAM, like the NDL1 protein localization, is limited to the organ primordium domains, which are situated at the periphery of all SAMs, and excluded from the central meristem zone, which is marked by *WUSCHEL* and *CLAVATA3* expression. Studies on *yabby* mutants have shown that YABBY proteins regulate growth, partitioning of the SAM and phyllotaxis [53], again like NDL proteins. Interestingly, in our search for NDL1 interactors we found YAB2 [56], and it is plausible that NDL proteins are part of the missing regulatory link between *AG* and *WUSCHEL,* and involved in the process of SAM maintenance and termination.

NDL proteins regulate the basipetal stream of auxin transport in roots [40] and stems (Fig 7). Interestingly, both up- and downregulation of all of the *NDL* members cause similar phenotypes, i.e., an increase in auxin transport and ectopic shoot formation. In contrast, DR5-GUS/GFP expression in the stem showed a direct correlation with the expression level of *NDL1* (Fig 7). This result suggests that NDL proteins may have an immediate/direct effect on the expression of auxin signaling components in stems, and their effect on polar auxin transport may be mediated by a possible connection with a secondary messenger such as *MAX2*, which regulates basal PIN1 localization in xylem parenchyma cells. We speculate that alterations in NDL levels induce an increase in basipetal auxin transport, which allows auxin to flow unimpeded down the stem, resulting in the depletion of auxin (Fig. 7B-E), and reduced auxin signaling at the node. This may activate the axillary meristems in the case of the *ndlM* mutant and cause abnormal silique development, and phyllotaxy in the case of *NDL1* over-expression.

Strigolactone metabolism, perception, and signaling is regulated by the α/β hydrolase fold-containing proteins (designated here as SLBPs). The SLBP functional homologs are: DAD2 in petunia, *Os*D14, in rice, and *At*D14 in *Arabidopsis* [57]–[59]. Strigolactones bind to SLBPs and promote subsequent interactions with the F-box protein MAX2. These interactions activate MAX2, which is a Skp-Cullin-F-box complex component, and this complex triggers the degradation of yet unidentified target proteins; downstream signaling results in lateral bud inhibition [60].

NDL1 is also an α/β hydrolase fold-containing protein but lacks the conserved catalytic triad (Ser-His-Asp) present in other strigolactone-interacting proteins. Although the catalytic triad is missing, the NDL1 protein model has a catalytic pocket and an overlying hydrophobic patch/flap that covers this pocket (see Fig. 1E [40]).

*NDL1* and *AGB1* to some extent also regulate auxin-directed organ formation by regulating the expression of *MAX2* through feedback regulation of *NDL1* and *MAX2* expression. Excess *NDL1* suppresses *MAX2* expression, and when *MAX2* is absent, the *NDL1* expression level decreases. AGB1 is also required in this process, but it functions in an *NDL1*-dependent manner (Fig. 8A and B). The threshold amount of NDL is critical; we speculate that NDL, a protein similar to SLBP, competes with SLBP for strigolactone binding and MAX2 activation, which in turn negatively regulates PIN1 levels and auxin transport.

**Figure 9:**
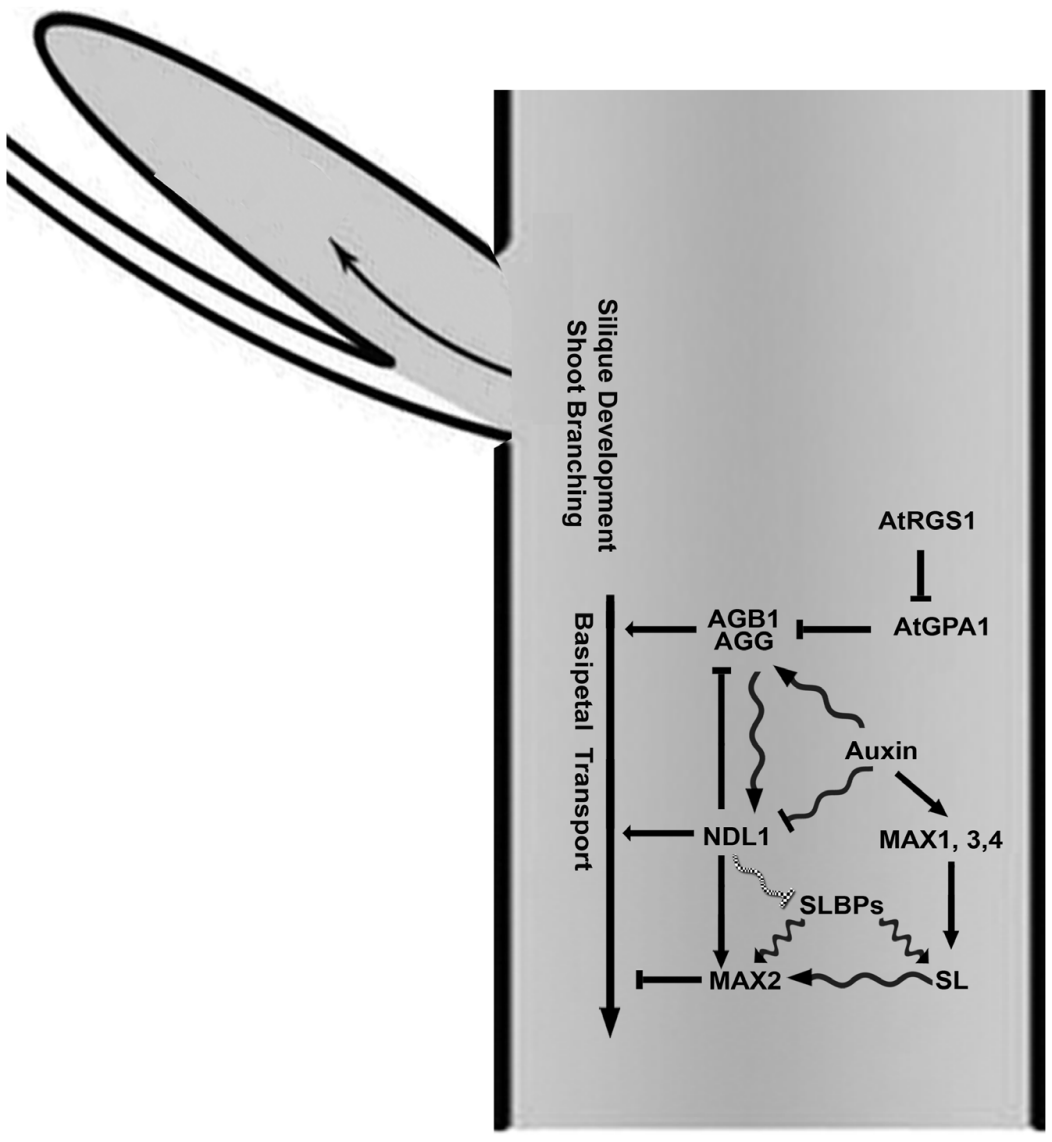
Proposed model for NDL function in the meristem and inflorescence stems. The connection between RGS1, GPA1 and AGB is based on data shown in this publication and previously published studies [83]–[86]. NDL1 and AGB1 are positive regulators of basipetal auxin transport (Fig 7A). They also function in auxin-regulated organ formation (Fig 1C and 3) and lateral meristem formation (Figs 2, 5, and 6) by establishing and/or maintaining auxin maxima (Fig 7B to I). Auxin positively regulates *AGB1* expression, whereas it has a negative effect on NDL1 stability [40]. Furthermore, auxin increases strigolactone biosynthesis, which subsequently activates MAX2 via SLBPs [5]. Together, NDL1 and AGB1 regulate *MAX*2 expression (Fig. 8). The scheme does not illustrate the actions of the three NDL proteins. Genetic interactions are represented by straight arrows, biochemical interactions are represented by wavy arrows, and a proposed interaction is represented by a wavy hatched arrow.

Fig. 9 illustrates the salient points from this work. AGB1 and NDL1 both directly or indirectly increase auxin transport, but the amount of NDL1 with respect to a threshold is critical. AGB1 and auxin control the stability of NDL1, and *AGB1* expression is regulated by auxins [40]. *NDL1* and *AGB1* in turn also regulate *MAX2* expression. Therefore, we postulate that there is a feedback loop between AGB1, NDL1, auxin and *MAX2*. The observation that reduced expression of *NDL* genes rescues some of the branching phenotypes resulting from the loss of AGB1 suggests that NDL1 attenuates some AGB1 function.

NDL proteins are likely the *Arabidopsis* orthologs of mouse NDRG1. NDRG1 interacts with AGB1 and AGG2 [40], suggesting a conserved and ancient function. Human orthologs to NDL proteins and mouse NDRG1 are among the few well-documented metastasis suppressors and are being used as possible cancer therapeutics [61]–[69]. NDRG1 is a novel effector for the small GTPase Rab4a and is important in recycling E-cadherin in proliferating cells [70], which provides insight into the metastasis mechanism. By analogy, small GTPase-mediated trafficking of PIN proteins is critical for auxin transport and the consequent location and size of auxin maxima [22], [23], [28], [71]–[75]. Pharmacological and genetic interference with the *Arabidopsis* ARF GEF GNOM leads specifically to apical localization of basal cargoes such as PIN1 [74], [76]. Auxin, through action of its cognate receptor AUXIN BINDING PROTEIN 1, coordinately activates two Rho GTPases, ROP2 and ROP6, within distinct domains of the membranes in a PIN1-dependent manner [77]. Other regulators of PIN protein endocytic recycling include other ARF GEFs (BEN/MIN7) [78] acting at the early endosome, the ARF GAP VAN3 [79], the coat protein clathrin, the actin cytoskeleton, and, indirectly, microtubules [71], [72], [76], [80]. Recently MAX2-mediated strigolactone signaling was found to trigger PIN1 depletion from the plasma membrane of xylem parenchyma cells in stems. This effect depended on clathrin-mediated membrane trafficking [45].

NDL proteins, in complexes with AtRGS1, AGB1 and other interacting proteins such as SYNTAXIN 23, may regulate the vesicular recycling of auxin transport facilitator proteins either directly or indirectly via crosstalk with MAX2.

It is postulated that auxin-regulated strigolactone biosynthesis is a conserved component of auxin-mediated branch inhibition and that auxin and strigolactone signaling may participate in an interlocking feedback loop that involves interplay with additional stimuli to precisely control branching in plants [13]. The highly branched *max* mutants have increased auxin transport capacity in the main stem resulting in increased bud outgrowth [10]. We previously showed that 1) levels of *NDL1* and *AGB1*, like *MAX* genes, are regulated in an auxin-dependent manner, 2) the NDL1-AGB1 signaling mechanism contains feedback loops in roots, 3) NDL promotes basipetal auxin transport in roots, and 4) NDL1 steady-state levels are negatively regulated by auxin [40]. We postulate that any alteration in NDL levels in stems results in alterations in auxin transport capacity, as occurs in the *max* mutants.

In summary, we described an aerial tissue function of NDL proteins as regulators of SAM formation. In this role, NDL proteins restrict proliferative cell division at the SAM and later, in due course of development, promote normal terminal differentiation of the floral meristem. The mechanism of this process involves the regulation of basipetal inflorescence auxin transport and local auxin gradients in the stem, and it may indirectly affect downstream inhibitory strigolactone signaling components.

## Materials and Methods

### Plant Material

Two independent transcript-null alleles for *NDL1 (ndl1-1* and *ndl1-2)* isolated from a T-DNA insertion population (ABRC) did not display obvious developmental defects, and insertion alleles for the other two homologs of *NDL1* (*NDL2* and *3*) were not null mutants. Therefore, a microRNA approach was taken. All the *NDL* genotypes, vectors, and primers were previously described [40]. *NDL* expression levels in the microRNA lines is also described in [40].

### Basipetal Inflorescence Stem Auxin Transport Assay

Basipetal auxin transport measurements in inflorescence stems were performed in various genetic backgrounds as described by Lewis and Muday [81]. Plants were grown in soil until the inflorescence stems were longer than 10 cm (∼thirty days). Inflorescence stems were excised at 2 cm and 4.5 cm from the apex and transferred to 20 µl of liquid [^3^H]-IAA (from Amersham Biosciences, 100 nM of 20–40 Ci mmol ^−1^) in an inverted orientation for 18 h. A 5 mm section of the shoot above the basal excision was assayed for radioactivity by scintillation counting. Control experiments with the base of the wild-type Col-0 inflorescence stem were used to measure background IAA movement [81]. The mean ± SEM values are based on at least five independent trials with each involving more than 10 shoots per genotype. Student's *t* tests are based on differences between wild type and the indicated genotypes. A confidence level of P<0.05 is indicated by an asterisk.

### Microscopy

Brightfield microscopy was performed using a Nikon inverted microscope (DIAPHOT-TMD; Nikon, Tokyo, Japan). Fluorescent protein fusions were analyzed using an Olympus XI81 inverted microscope (Olympus America Inc., Melville, NY). The GUS-stained SAMs were embedded in JB-4 plastic and sectioned as described previously [40]. Phloroglucinol staining was used to stain lignin [82].

For field emission scanning electron microscopy, samples were fixed (in 3.5% paraformaldehyde) and dehydrated in a series of increasing ethanol concentrations (30%, 50%, 75%, and 100%). Samples were critical point-dried in a Tousimis Samdri-795 critical point dryer (Tousimis Research Corporation, Rockville, MD) with liquid CO_2_ as the transitional fluid.

Specimens were mounted on aluminum stubs with carbon adhesive tape and sputter-coated with 10 nm of gold/palladium (60∶40) using a Hummer X sputter coater (Anatech USA, Union City, CA). Samples were imaged at 5 kV using a Zeiss Supra 25 field emission scanning electron microscope (Carl Zeiss SMT, Inc., Peabody, MA).

For assessment of DR5:GUS and DR5:GFP expression, apical regions (1 cm to 4 cm from the top) and stems (1 cm to 5 cm long) were hand-sectioned using a double-edged blade. For DR5:GUS analysis, the sections were GUS stained, cleared and mounted in chloral hydrate:glycerol:water (8∶3∶1). For DR5:GFP analysis, the sections were directly mounted in 10% glycerol. The sections were visualized, and images were taken using a Primo Star (Zeiss) microscope for GUS and a Nikon microscope for GFP. Analyses were performed on five to six-week-old *Arabidopsis* plants grown in pots (24 °C, 16 h/8 h light/dark).

### Quantitative real time (qRT) PCR

RNA was isolated from the flowers of various genotypes, and first strand cDNA was synthesized. qRT-PCR was performed in triplicate with three biological replicates. These reactions, the qRT-PCR details, *NDL1* primers, and the reference gene (*ACTIN2*) primer sequences were previously described [40] *MAX2* qRT-PCR primers were also designed using GenScript Real-time PCR (TaqMan) Primer Design software (http://www.genscript.com/ssl-bin/app/primer) as previously described [40]. Primers used to amplify MAX2 were: MAX2 qRT For (GACCTCCCTGACGTCATCTT) and MAX2 qRT Rev (GACGAGGGAGAGAGAGTTGC).

### Accessions

Sequence data from this article can be found in the GenBank/EMBL data libraries under the following accession numbers: At4g34460 (*AGB1*), At3g63420 (*AGG1*), At3g22942 (*AGG2*), At3g26090 (*RGS1*), At5g56750 (*NDL1*), At5g11790 (*NDL2*), At2g19620 (*NDL3*), At3g18780 (*ACTIN2*), and At2g42620 (*MAX2*).

## Supporting Information

File S1 **Contains Figure S1**. Asymmetrical localization of NDL1-GUS in cotyledons from dark- and light-grown plants. **Figure S2**. Ectopic over-expression of *NDL1* resulted in abnormal development of vegetative and reproductive whorls. **Figure S3**. Ectopic over-expression of *NDL1* resulted in atypical flowers with abnormal numbers of flower whorls. **Figure S4**. Leaves from various independent *ndlM* knockdown lines showing lamina defects. **Figure S5**. Histology of the stem vasculature of wild-type Col-0 and *ndlM* mutants expressing *DR5-GUS*.
(PDF)
