# Supplementary material for: N-MYC DOWN-REGULATED-LIKE Proteins Regulate Meristem Initiation by Modulating Auxin Transport and MAX2 Expression
Source: PLoS One. 2013 Nov 4;8(11):e77863. doi: 10.1371/journal.pone.0077863 (PMC3817199; doi:10.1371/journal.pone.0077863)
Supplement: File S1 — Contains Figure S1. Asymmetrical localization of NDL1-GUS in cotyledons from dark- and light-grown plants. Figure S2. Ectopic over-expression of NDL1 resulted in abnormal development of vegetative and reproductive whorls. Figure S3. Ectopic over-expression of NDL1 resulted in atypical flowers with abnormal numbers of flower whorls. Figure S4. Leaves from various independent ndlM knockdown lines showing lamina defects. Figure S5. Histology of the stem vasculature of wild-type Col-0 and ndlM mutants expressing DR5-GUS. (PDF) [file pone.0077863.s001.pdf]

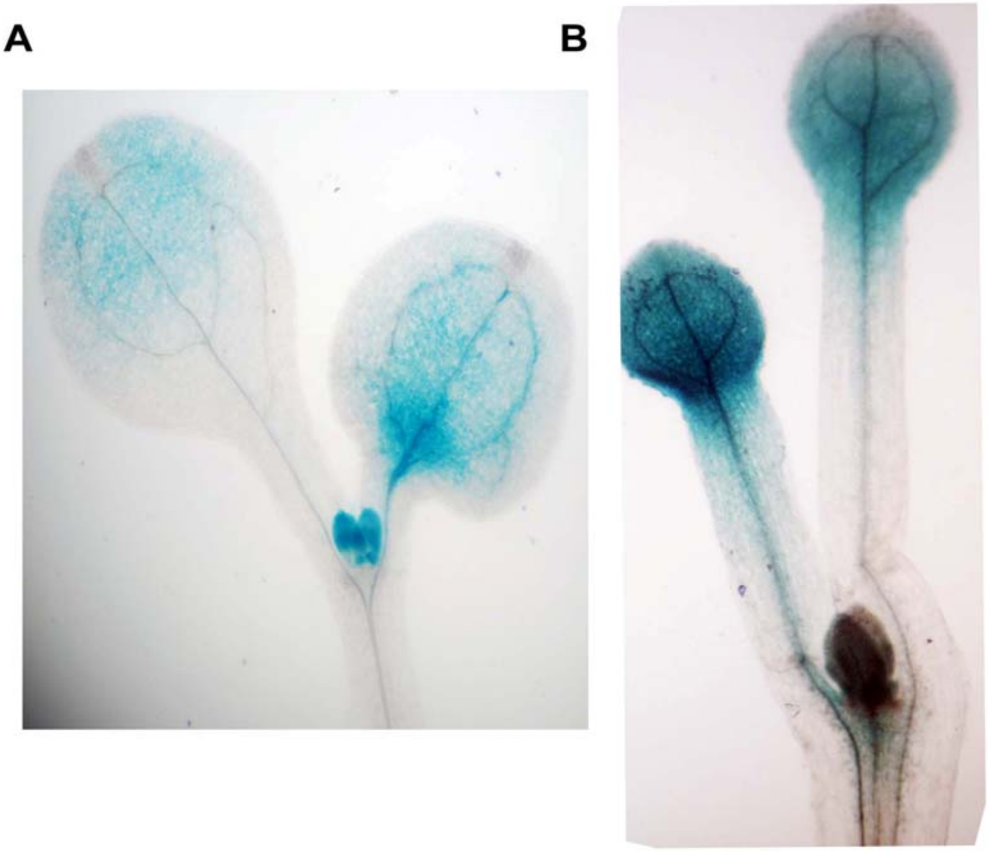

Figure S1. **Asymmetrical localization of NDL1-GUS in dark- and light-grown cotyledons.**

- **A.** NDL1-GUS Localization analysis in 3-4 days- old light- grown seedling. Uneven localization of NDL1- GUS staining in cotyledons. Right cotyledons staining darker than the left one.
- **B.** Seven-day-old dark grown seedling, it has a dark stained lagging, in terms of expansion (left) and comparatively lightly stained leading cotyledon..

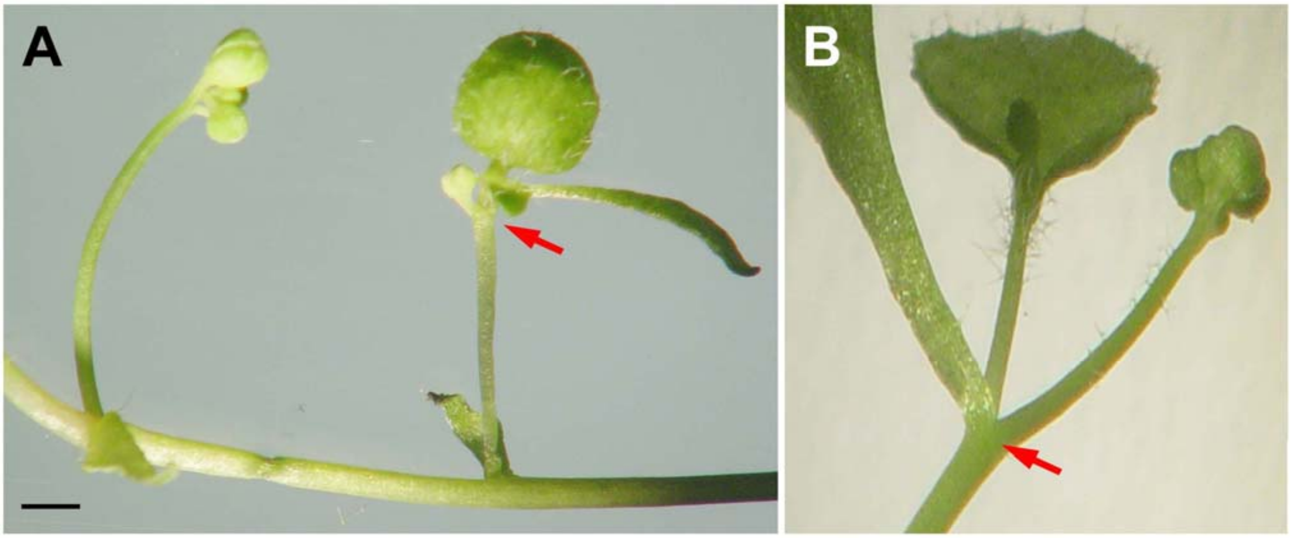

Figure S2. Ectopic over-expression of *NDL1* resulted in abnormal development of vegetative and reproductive whorls.

A-B. A single node bears a leaf, cauline leaf and flowers. Arrow marks the point of origin of these organs.

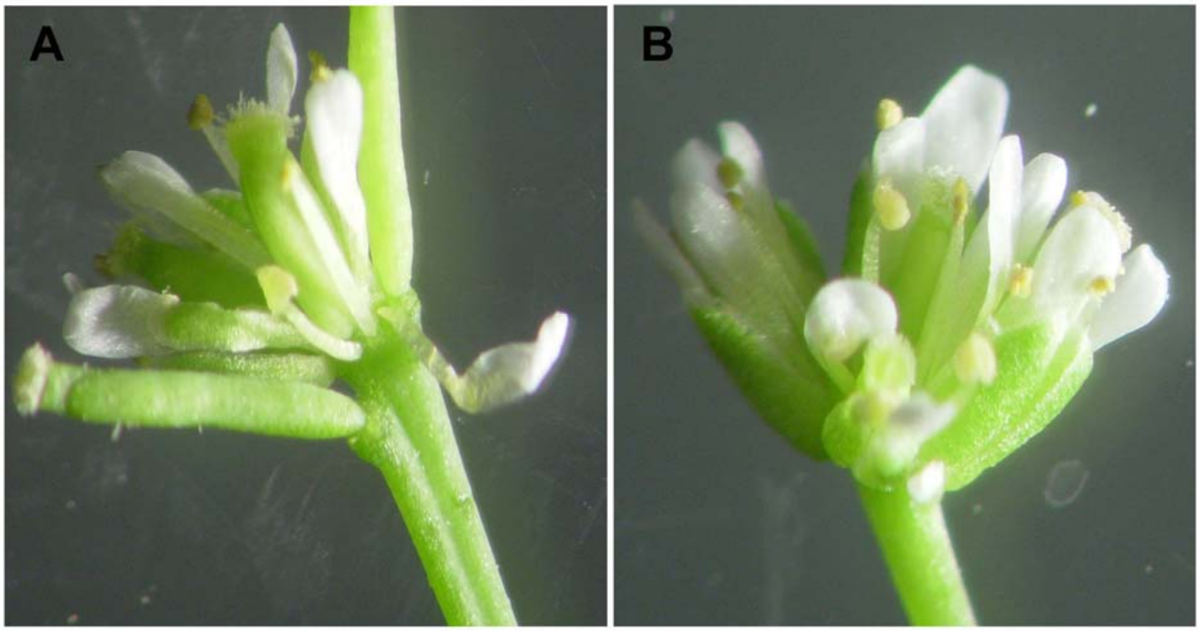

Figure S3. Ectopic over-expression of *NDL1* resulted in atypical flowers with abnormal number of flower whorls.

- **A.** Flower with four pistils and multiple petals pedicel appears fused structure.
- **B.** Single pedicel with numerous sepals, petals, pistils and stamens.

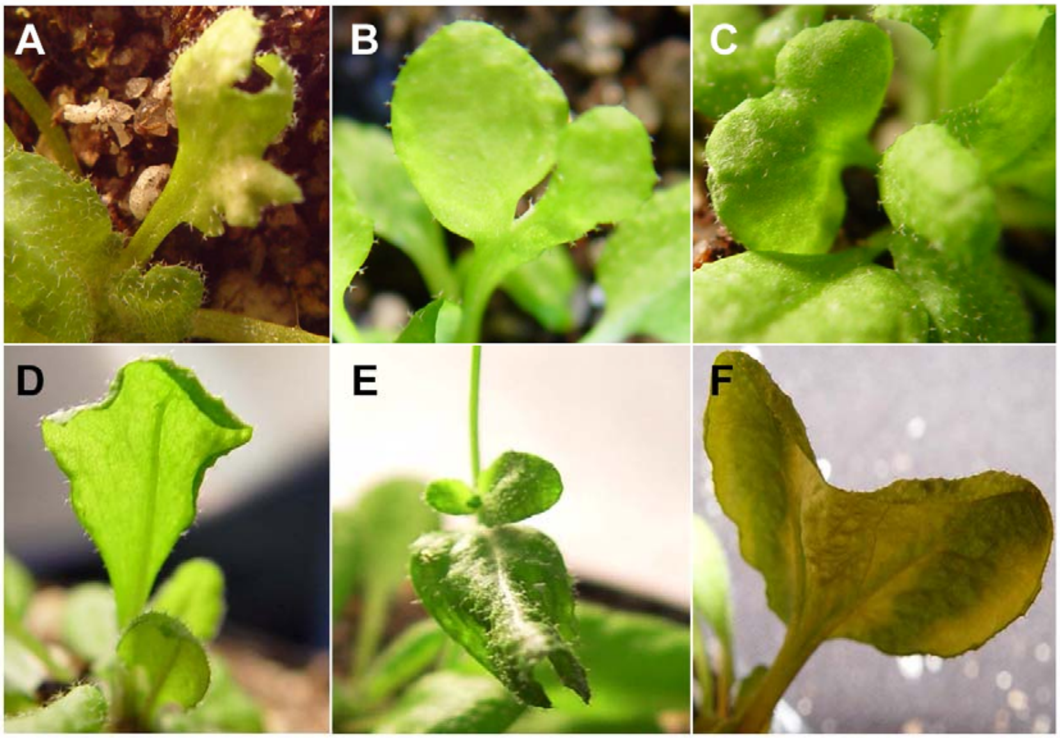

Figure S4. Leaves from various independent *ndlM* knock-down lines showing lamina defects.

A. Leaf showing highly serrated margin.

B. Bi-lobed leaf.

C. Expanded heart shaped leaf.

D-E. Expanded and folded leaf.

F. Heart shaped leaf showing two independent midveins to two lobes.

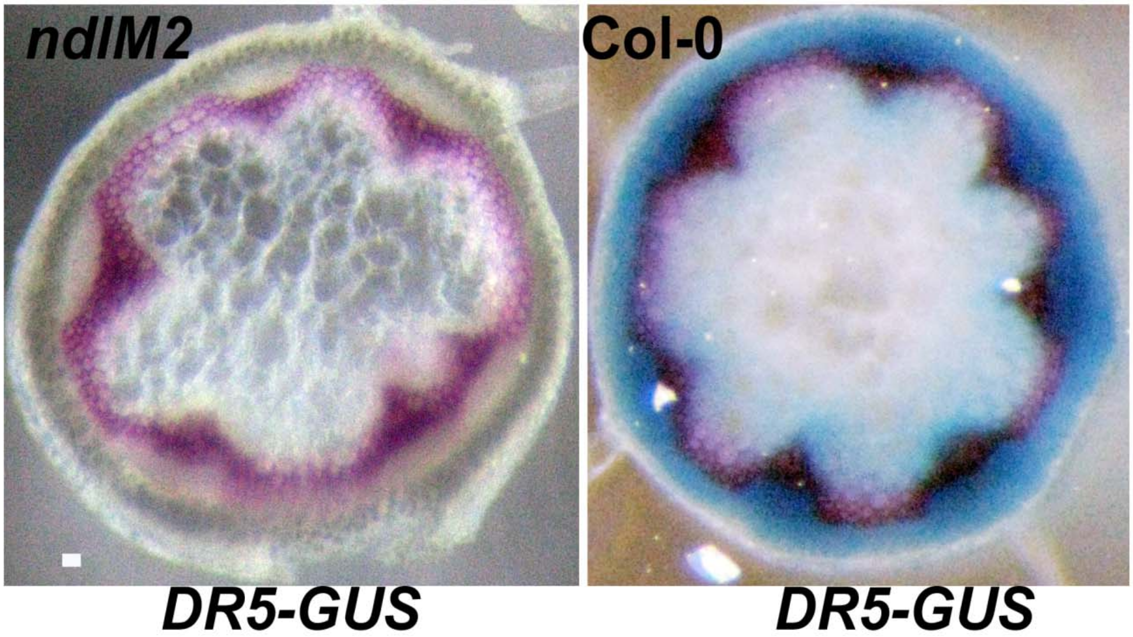

Figure S5. Histology of the stem vasculature of wild-type Col-0 and *ndlM* mutant expressing *DR5-GUS.*

Phloroglucinol staining was used to stain amount of vasculature present. Sections already stained with GUS stain were stained with phloroglucinol stain and responsible for double staining in sections.
